# Editorial: Recent Advances of Evidence-Based Neuropsychology

**DOI:** 10.3389/fpsyg.2021.740450

**Published:** 2021-08-11

**Authors:** Guangyu Zhou, Xi Wang

**Affiliations:** School of Psychological and Cognitive Sciences and Beijing Key Laboratory of Behavior and Mental Health, Peking University, Beijing, China

**Keywords:** review, evidence-based neuropsychology, recent advances, mental disorder (disease), chronic illness

Neuropsychology stands at the intersection of brain and behavior. This distinctive position gives it the unique opportunity to provide insights into the pathophysiological mechanisms underlying some physical and mental health disorders that plagues human society for centuries. Based on the extending knowledgebases in the broad spectrum of neuroscience and psychology, the evidence-based neuropsychology (EBN) is an emerging area over the past years, focusing on translating scientific advances into daily clinical practice. As many questions in this area remains inconclusive, the goal of this Research Topic is to present a diversified sample of the most recent advances in the field and to foster promising ideas for future study.

Chronic diseases such as diabetes have become the predominant challenge to global health in the twenty-first century, accounting for millions of deaths annually worldwide (Yach et al., 2004; Bauer et al., 2014). The success of controlling chronic diseases lies greatly in patients' self-regulation on their health behavior such as eating habits and exercise (Richardson et al., 2019). To promote well-controlled self-management of patients, Zhao et al. highlighted the importance of addressing deficit in executive function resulted from diabetes. Without proper intervention, the decline of executive function may subsequently lead to slack diabetes management, thereby fostering a vicious cycle.

In addition to the physical diseases, the mental health also closely relates to health behavior (Glanz et al., 2008). Therefore, the modification of health behavior offers a viable strategy to treat mental disorder effectively and equitably. By systematically reviewing published literatures, Chen et al. confirmed the effectiveness of exercise intervention, one of the most investigated health-behavior-based intervention, to common neuropsychological disorders, and disentangled the possible psychological and physiological mechanisms underlying the exercise-induced benefits.

Apart from long-existing health problems, novel health problems emerge along with the advancement of technology. Li et al. explored Internet addiction, one of such new health threats. Their results indicate that exercise-based interventions are effective in treating not only the “traditional” health problems, as shown by Chen et al., but also health risks in modern days.

Instead of focusing on a specific type of diseases, Zhang et al. adopted a broader view, identifying top 100 articles in neuropsychology with most citations using the bibliometric analysis. The results highlight the emphasis of original and innovative ideas in this area, as indicated by the observation that original studies gained more citations than review papers.

The articles in this Research Topic are widely positioned across the field of evidence-based neuropsychology. They reviewed some progress made in evidence-based neuropsychology and illustrated various facets of the field. These articles are presented with the hope to inform readers with the latest advancement in the area and to enlighten future research.

## Author Contributions

GZ conceived the idea and structure of the manuscript. XW drafted the manuscript. All authors contributed to the article and approved the submitted version.

## Conflict of Interest

The authors declare that the research was conducted in the absence of any commercial or financial relationships that could be construed as a potential conflict of interest.

## Publisher's Note

All claims expressed in this article are solely those of the authors and do not necessarily represent those of their affiliated organizations, or those of the publisher, the editors and the reviewers. Any product that may be evaluated in this article, or claim that may be made by its manufacturer, is not guaranteed or endorsed by the publisher.
